# Improving Yellow Mealworm (*Tenebrio molitor*) Utilization with Sodium Butyrate in Nile Tilapia Diets: Effects on Growth Performance, Intestinal Histology, Antioxidative Response, and Blood Biomarkers

**DOI:** 10.1155/2024/2442308

**Published:** 2024-03-26

**Authors:** Fify F. El-Desouky, Mostafa A. Ibrahim, Ibrahim M. Abd El-Razek, El-Said M. El-Nabawy, Asem A. Amer, Amr I. Zaineldin, Mahmoud S. Gewaily, Mahmoud A. O. Dawood

**Affiliations:** ^1^Animal Production Department, Faculty of Agriculture, Kafrelsheikh University, Kafr El-Sheikh, Egypt; ^2^Department of Entomology, Faculty of Agriculture, Kafrelsheikh University, Kafrelsheikh, Egypt; ^3^Department of Fish Nutrition and Feed Technology, Central Laboratory for Aquaculture Research, Agricultural Research Center, Abbassa, Abo-Hammad 44662, Sharqia, Egypt; ^4^Agriculture Research Center, Animal Health Research Institute (AHRI-DOKI), Giza, Egypt; ^5^Department of Anatomy and Embryology, Faculty of Veterinary Medicine, Kafrelsheikh University, Kafr El-Sheikh, Egypt; ^6^The Center for Applied Research on the Environment and Sustainability, The American University in Cairo, Cairo 11835, Egypt

## Abstract

Yellow mealworm (*Tenebrio molitor*) meal was introduced to aquafeed as a suitable protein source to replace fish meal (FM) and soybean meal and, thereby, consistent aquaculture production. However, mealworms should be added at adequate levels due to the presence of antinutritional factors such as chitin. Consequently, sodium butyrate (SB) is suggested to improve feed quality and ensure aquatic animals' productivity and welfare. In this study, parallel with the protein source (*T. molitor* meal or FM), dietary supplementation of SB (1 g/kg) is involved as a factor in the 2 × 2 factorial study. The first and the second diets were formulated using FM as a protein source with or without SB, while the third and fourth diets were prepared by replacing FM with *T. molitor* meal with or without SB supplementation. After 60 days, fish fed with FM or *T. molitor* and SB showed improved final body weight and weight gain, while those fed with *T. molitor* without SB had a reduced protein efficiency ratio. Histological analysis revealed that dietary SB improved intestinal histological features by increasing the height and branching of intestinal villi and immune cell infiltration near intestinal crypts in Nile tilapia-fed FM or *T. molitor*. Furthermore, fish-fed FM or *T. molitor* and SB had higher Hb, red blood cells, PCV, total protein, and globulin levels than fish-fed respective test diets without SB supplementation. Dietary SB addition to FM or *T. molitor*-based diets also significantly enhanced blood lysozyme and phagocytic activities, catalase, superoxide dismutase, glutathione peroxidase, and reduced MDA levels. Our results demonstrate that *T. molitor* meal can replace FM without compromising Nile tilapia's growth performance and health status. Additionally, SB supplementation improved *T. molitor* meal utilization by Nile tilapia, thereby significantly enhancing the growth, digestion capacity, intestinal histological features, and antioxidative and immune responses. Consequently, dietary *T. molitor* meal reduces the reliance on FM and improves the sustainability and efficiency of Nile tilapia production.

## 1. Introduction

The aquaculture sector has sustained humanity with safe and delicious animal protein for a long time [1]. Nonetheless, the continuous rise in human consumption requires more supply of seafood protein [2]. The expansion of aquaculture depends on the aquafeed industry since feed constitutes more than 60% of the total running cost for production [3]. The aquafeed industry uses high-quality protein sources to provide aquatic animals with the energy required for metabolic and physiological functions [4, 5]. Traditionally, fish meal (FM) and plant ingredients (e.g., soybean meal, corn gluten, wheat middlings, sunflower meal, etc.) are used to prepare nutritionally balanced feed formulations [6, 7]. The huge consumption of FM in feed production caused severe shortages and coincided with a dramatic price increase [8, 9]. However, by 2050, the available byproducts of fisheries and processed aquatic animals will not be enough to afford aquafeed requirements [10]. Subsequently, appreciated efforts were investigated to replace FM with plant protein sources, which showed potential possibilities depending on feeding behavior and environmental conditions [11, 12]. However, using plant ingredients is also inevitable due to the high pressure on land and water resources, as well as the low content of essential amino acids and nutrients compared to FM [13]. Recently, the incorporation of insect meals has been approved as a suitable protein content for the sustainable aquafeed industry [14].

Insect meals are introduced to the aquafeed industry as a low-cost and affordable animal protein source that may substitute FM and soybean meal [15]. The yellow mealworm (*Tenebrio molitor*) is an optimistic insect known for its fast growth and reproduction and can efficiently utilize plant byproducts and grains [16]. Indeed, yellow worm meal has been allocated as a sustainable alternative protein source with a low environmental footprint [17]. Yellow worms contain suitable protein (47%–63%), amino acids, and lipid (31%–41%) contents that satisfy aquatic animals' needs [18]. In this context, the inclusion of yellow worms in aquafeed has been reviewed recently, as stated by Zhang et al. [19], Li et al. [20], and Tran et al. [21]. Yellow worms replaced up to 75% of FM without interrupting the growth performance and health status of several carnivorous, omnivorous, and herbivorous fish species [22]. However, a high inclusion rate of yellow worm meal in aquafeed may interrupt intestinal health and lead to low feed utilization, irregular metabolic rate, and retarded growth performances [23, 24]. Thus, it is crucial to investigate several feeding strategies to accommodate the optimum inclusion levels of insect meals without affecting fish performances [1, 25].

The gastrointestinal tract of fish is one of the main gates for the attack of harmful invaders on the entire body [26]. Thus, protecting fish's intestinal health and functionality is vital since unreasonable aquafeed formulation may interrupt intestinal health and cause severe drawbacks [27]. Furthermore, there must be insurance to increase the palatability and digestibility of yellow worm meal to afford fish with nutritionally balanced feeds [25]. Organic acids have been successfully applied in aquaculture for their safety and efficacy as growth and feed digestion enhancement ability [28, 29]. Sodium butyrate is a short-chain fatty acid applied in aquaculture as a functional additive [30]. The sodium butyrate salt is formed by the conjunction of butyric acid and sodium, resulting in a stable form with less intensity than butyric acid [31]. Hence, it is the most recommended form of organic acids since sodium butyrate facilitates the availability of nucleotides and amino acids through the intestines [32]. Further, sodium butyrate provides sufficient energy for the intestinal epithelial cells, thereby enhancing the absorption of digested nutrients to the fish's entire body [33]. Concurrently, dietary sodium butyrate enhanced the zootechnical, feeding, and health performances of several fish species (reviewed by Fabay et al. [31] and Abdel-Latif et al. [29]).

Widely, Nile tilapia (*Oreochromis niloticus*) culture has increased substantially due to its high food quality, suitability for intensive farming, and encouraging market value [34, 35]. The inclusion of yellow worm meal in tilapia feed has been investigated without negative impacts on growth performance, digestion, and well-being. In this regard, Tubin et al. [36] and Anany et al. [37] stated that yellow worm meal could be included in tilapia diets up to 10% without affecting growth performance. Further, dietary yellow worm meal could replace up to 50% of FM or soybean meal, as declared by Sánchez–Muros et al. [38]. It is worth noting that lately, Anany et al. [37] concluded that dietary *Saccharomyces cerevisiae* improved the acceptability of Nile tilapia to yellow worm meal inclusion. The results indicated improved growth performance, feed digestion, intestinal health, and immune and antioxidative responses. In this sense, this trial aimed to improve yellow worm utilization by adding sodium butyrate through evaluating the growth performance, feed digestion, intestinal health, and biochemical parameters.

## 2. Materials and Methods

### 2.1. Diet Formulation

Four test diets were prepared to fulfill the requirements of Nile tilapia by following the NRC [39] (Table 1). The first and the second diets were formulated using FM and soybean meal as protein sources with or without sodium butyrate (SB; AVITASA, Spain) addition. At the same time, the third and fourth diets were prepared by replacing FM with *T. molitor* meal with or without SB supplementation. *T. molitor* meal was cultured at the Department of Entomology, Faculty of Agriculture, Kafrelsheikh University, and its chemical composition was checked by following the AOAC [41]. Dietary SB was included in the second and fourth diets at 1 g/kg diet by following Dawood et al. [42]. Yellow corn, corn gluten, wheat bran, rice bran, and wheat flour were added to the diets to fulfill the carbohydrate requirements. Fish oil and corn oil were used as lipid sources. Vitamin and mineral mixture, dicalcium phosphate, and vitamin C were also included to balance the micronutrient needs. All ingredients were finely grounded and were thoroughly well mixed using the laboratory food mixer (El-Adl™, Tanta, Egypt). The SB was added to the second and fourth diets, and all ingredients were remixed. All ingredients were mixed with water at 35%–40%, then pellets (2 mm) were produced using the laboratory pelletizing machine. Pellets were broken to proper sizes for fish, then dried, and finally collected and kept in plastic bags until use. Storage of formulated test feeds was in a freezer at −20°C. The chemical composition of the test diets was checked by following the AOAC [41].

### 2.2. Feeding Trial and Termination

Homogenous sizes of mono-sexed Nile tilapia juveniles were collected from a hatchery located on the International Road to Baltim City, Kafrelsheikh, Egypt. Then, fish were kept in suitable plastic tanks provided with a source of aeration and transported gently to the Laboratories and Greenhouses for the Faculties of Agriculture and Veterinary Medicine, Kafrelsheikh University, Egypt. Fish were randomly distributed on arrival in 12 prior prepared glass aquaria (100 L). All aquaria were supplied with continuous aeration via electrical aerators. Free chlorine water was added to all aquaria, and half of the water was exchanged every 2 days. For 15 days, all fish were offered the basal diet at 3% of their body biomass. After the adaptation period, all fish with an average initial weight of 6.05 ± 0.03 g/fish were redistributed at 20 fish per aquarium. Each experimental diet was offered to three aquaria, and the trial was done in 12 aquaria (triplicate). The test diets were offered to the fish up to the satiation level twice daily at 08:00 am and 2:00 pm. Fish were observed during the feeding times, and when fish stopped consuming pellets, the feed was terminated, and the amount of feed intake was reported. Half of the water was exchanged with fresh, free chlorine water during the feeding trial (60 days). Water quality traits were regularly monitored during the trial and kept at 7.61 ± 0.44, 6.11 ± 0.28 mg/L, 27.87 ± 0.34°C, 6.11 ± 0.28 mg/L, and 0.01 ± 0.001 g/L for dissolved oxygen, pH, temperature, and total ammonia nitrogen, respectively.

After 60 days, the fish were starved for 24 hr before the final sampling. All fish were anesthetized with tricaine methane sulfonate (MS-222) (100 mg/L), and their individual weight and numbers in each aquarium were recorded. In parallel, three fish per aquarium were randomly collected for blood collection and dissection. Blood was obtained from the caudal vein using 5 mL gauge syringes and divided into two portions. Using EDTA-heparinized tubes, the first portion of blood was kept to perform fresh blood hematological analysis. The other blood portion was kept in nonheparinized tubes to allow serum separation. Serum was collected after keeping tubes in a refrigerator at 4°C and then centrifuged at 3,000 rpm for 15 min at 4°C (SCILOGEX, Model: DM0412, USA). The serum was then separated and kept at −20°C for further analysis. Meanwhile, fish were dissected to have the intestine and liver organs which were fixed in 10% neutral buffered formalin after dissection.

### 2.3. Growth Performance and Feed Utilization

The growth performance and survival traits were calculated using the following equations:

(1) $$\begin{array}{c} \text{Weight gain }\left(\text{WG}\right)=100\times \frac{\left(\text{FW}\left(\text{g}\right)-\text{ IW}\left(\text{g}\right)\right)}{\text{ IW}\left(\text{g}\right)}, \end{array}$$

(2) $$\begin{array}{c} \text{Specific growth rate }\left(\text{SGR},\%\text{/day}\right)=100\times \frac{\left(\text{Ln FW}\left(\text{g}\right)-\text{ Ln IW}\left(\text{g}\right)\right)}{60\text{ days}}, \end{array}$$

(3) $$\begin{array}{c} \text{Survival (\%)}=100\times \frac{\text{final fish number}}{\text{initial fish number}}. \end{array}$$

In addition, the feed utilization was calculated using the following equations:

(4) $$\begin{array}{c} \text{Feed conversion ratio }\left(\text{FCR}\right)=\frac{\text{TFI}\left(\text{g}\right)}{\left(\text{FW}\left(\text{g}\right)-\text{ IW}\left(\text{g}\right)\right)}, \end{array}$$

(5) $$\begin{array}{c} \text{Protein efficiency ratio }\left(\text{PER}\right)=\frac{\left(\text{FW}\left(\text{g}\right)-\text{ IW}\left(\text{g}\right)\right)}{\text{ dry protein intake}\left(\text{g}\right)}, \end{array}$$

where IW: initial weight (g), FW: final weight (g), and TFI: total feed intake (g).

### 2.4. Histology Study

The obtained intestine and liver samples were moved from 10% neutral buffered formalin to 70% alcohol after 24 hr. The samples were then cleaned in xylene, impregnated, and embedded in paraffin wax after being dehydrated in an increasing graded series of ethanol [40]. Leica rotatory microtome (RM 20352035; Leica Microsystems, Wetzlar, Germany) was used to cut sections measuring 5 *µ*m, which were then placed on glass slides. Hematoxylin and eosin (H&E) staining was applied to the produced tissue sections [43]. Using a light microscope (Olympus, Tokyo, Japan), the stained sections were inspected. A light microscope (Leica DM500; Leica Microsystems, Heerbrugg, Switzerland) was used to view the stained sections.

### 2.5. Blood Analysis

#### 2.5.1. Hematology

Red blood cell (RBC), white blood cell (WBC) counts, and their derivatives were performed following the recommended procedure [44]. The hemoglobin (Hb) concentration was measured using a spectrophotometer (Model RA 1000, Technicon Corporation, USA) at 540 nm using the Blaxhall and Daisley [45] method, while the packed cell volume (PCV) was estimated using the microhematocrit method.

#### 2.5.2. Biochemical Traits

The procedures detailed by Doumas et al. [46] and Dumas [47] were used to determine the blood levels of albumins and total proteins. The difference in the total protein and albumin levels was employed to compute the globulin levels numerically. Using the RA-50 chemical analyzer (Bayer), serum levels of triglycerides, creatinine, total cholesterol, and aspartate aminotransferase (ALT) were determined. Ready-made chemical kits from Spinreact Co. Spain were used in accordance with the manufacturer's instructions.

#### 2.5.3. Immunity and Antioxidative Traits

A turbidimetric assay, based on the method of Ellis et al. [48], was used to analyze serum lysozyme activity. A standard suspension of 0.15 mg/mL of *Micrococcus lysodeikticus* (Sigma, USA) was prepared in 66 mM phosphate buffer (pH 6.0). Serum (50 *μ*L) was then added to 1 mL of the bacterial suspension, and the absorbance reduction was recorded at 30 s and 4.5 min intervals at 450 nm using a spectrophotometer (SHIMADZU UV-1600PC). One unit of lysozyme was defined as a reduction in absorbance of 0.001/min.

Leukocyte phagocytic function was assessed following the technique described by Cai et al. [49]. The number of leukocytes that engulfed bacteria was counted as percentages in relation to the total leukocyte number in the smear from the phagocytosis assay. The phagocytic activity and index were determined by Kawahara et al. [50]. Briefly, *Candida albicans* suspension (equivalent to 1 × 10^6^) was prepared and mixed with 100 *μ*L of a fresh blood sample and fetal bovine serum. Then, the solution was mixed and incubated at 37°C for 30 min; the mixture was centrifuged at 1,500 rpm for 10 min. Five microliters of resuspended cells were used for the blood smear. Phagocytic activity equals the percentage of phagocytic cells that engulfed yeast cells. The phagocytic index equals the total number of yeast cells phagocytized divided by the number of phagocytic cells.

The glutathione peroxidase (GPx), catalase (CAT), and superoxide dismutase (SOD) were detected using diagnostic reagent kits from Cusabio Biotech Co., Ltd. (China) in accordance with the manufacturer's instructions. The malondialdehyde (MDA) content was measured and reported in nmol MDA/mL [51]. In brief, 10% w/v serum samples were combined with 0.5 mL of 0.6% thiobarbituric acid and 1.5 mL of 1% H_3_PO_4_. The tubes were placed in a bath of hot water for 60 min. Following cooling in an ice bath, 2 mL of butanol was added, and the mixture was violently stirred for 20 s. A spectrometer (Lambda 2S, Perkin–Elmer Co.) was used to evaluate the wavelength (520 and 535 nm) of the organic layer post-centrifugation (3,000 rpm, 15 min).

### 2.6. Statistical Analysis

All data were tested for homogeneity of variance by Shapiro–Wilk and Levene tests, and normality was tested by Kolmogorov–Smirnov test. Data were analyzed as a two-way ANOVA (2 factorial design) using the general linear model procedure. The main protein sources (FM or *T. molitor*), dietary sodium butyrate (SB), and their interaction. Tukey's multiple comparisons test compared means when interactive effects differed significantly. Values have presented an average of three replicates. Significant differences (*P* < 0.05) between dietary protein sources (FM or *T. molitor*) and SB were evaluated by Tukey's test. However, in the case of nonsignificant interactions at (*P* > 0.05), the significance between dietary protein sources (FM or *T. molitor*) or SB was evaluated by *t*-test at (*P* < 0.05). All the statistical analyses were done via SPSS version 22 (SPSS Inc., IL, USA).

## 3. Results

### 3.1. Growth Performance

The growth performance, survival rate, and feed efficiency of Nile tilapia-fed FM or *T. molitor* with or without sodium butyrate (SB) supplementation are presented in Table 2. Marked interaction effects of protein source and dietary SB were seen on the FBW (*P*=0.013), WG (*P*=0.012), SGR (*P*=0.003), FCR (*P*=0.044), and PER (*P*=0.017) of Nile tilapia. Fish-fed FM and SB had higher FBW and WG than those fed FM without SB or *T. molitor* without SB. No significant effects were seen between the FBW and WG in the case of fish-fed FM with SB and fish-fed *T. molitor* with SB. The SGR was meaningfully higher in tilapia-fed FM with SB than those fed without SB or *T. molitor* with or without SB. Tilapia-fed *T. molitor* without SB had higher FCR and lower PER than the remaining groups. Under the current study conditions, no interaction effects for protein sources and dietary SB were seen on the feed intake and survival rate of Nile tilapia (*P* > 0.05).

### 3.2. Intestinal Histomorphometry and Liver Histology

**Figure 1:**
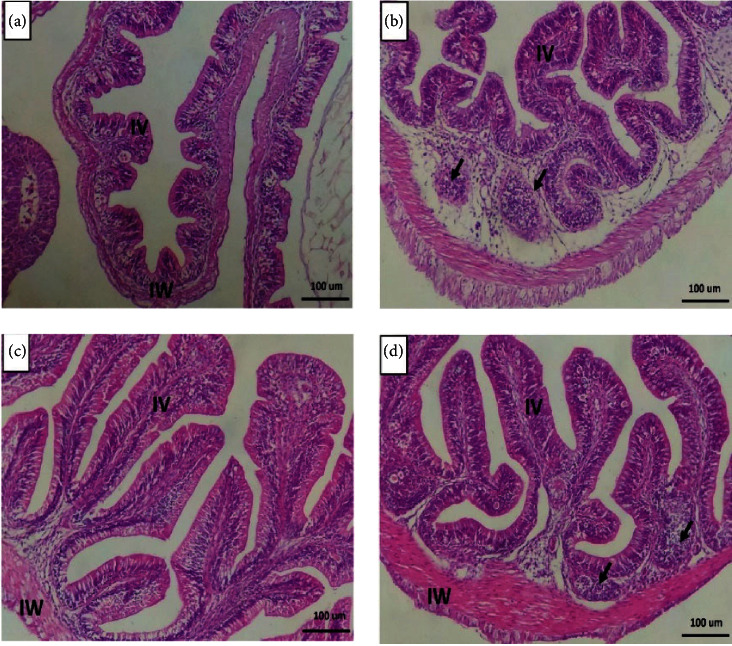
Histomicrograph showing the histological structure of the middle segment of Nile tilapia intestine in the fish meal (a), *T. molitor* (b), fish meal/sodium butyrate (c), and both *T. molitor*/sodium butyrate (d). The intestinal wall (IW) intestinal villi (IV) showed apparent growth, mainly in group (d), in addition to immune cell infiltration (black arrow) in groups (b) and (d). Stain H&E. Bar = 100 *µ*m.

In all groups, fish intestines had intact intestinal walls and villi structures (Figure 1(a)–1(d)). The intestinal wall comprises serosa in the outermost layer, tunica muscularis, propria submucosa, and tunica mucosa inside. The intestinal villi contains goblet cells grouped around a connective tissue core and simple columnar cells extending within the intestine's lumen. The appearance of the intestinal villi in fish-fed *T. molitor* and/or SB along the entire length of the intestine revealed a considerable increase in height and branching (Figure 1(b)–1(d)). Furthermore, the infiltration of immune cells was observed in the vicinity of the intestinal crypts of the intestine in the groups that were given *T. molitor* and SB with *T. molitor* only (Figures 1(b) and 1(d)).

The intestinal morphometrical indices of Nile tilapia-fed FM or *T. molitor* with or without SB supplementation are presented in Table 3. Marked interaction effects of protein source and dietary SB were seen on the morphometrical indices (villus height (*P*=0.021), villus width (*P*=0.043), crypt depth (*P*=0.003), muscular thickness (*P*=0.002), and goblet cell count (*P*=0.003)) detected (*P* < 0.05) in the middle intestines of Nile tilapia. Fish-fed FM or *T. molitor* and SB had higher villus height, width, crypt depth, muscular thickness, and goblet cell count than those fed FM or *T. molitor* without SB. The villus height, crypt depth, muscular thickness, and goblet cell count were significantly higher in tilapia-fed *T. molitor* and SB than in tilapia-fed FM with or without SB. Besides, the villus height, width, crypt depth, muscular thickness, and goblet cell count were significantly higher in tilapia-fed SB than in tilapia-fed without SB.

**Figure 2:**
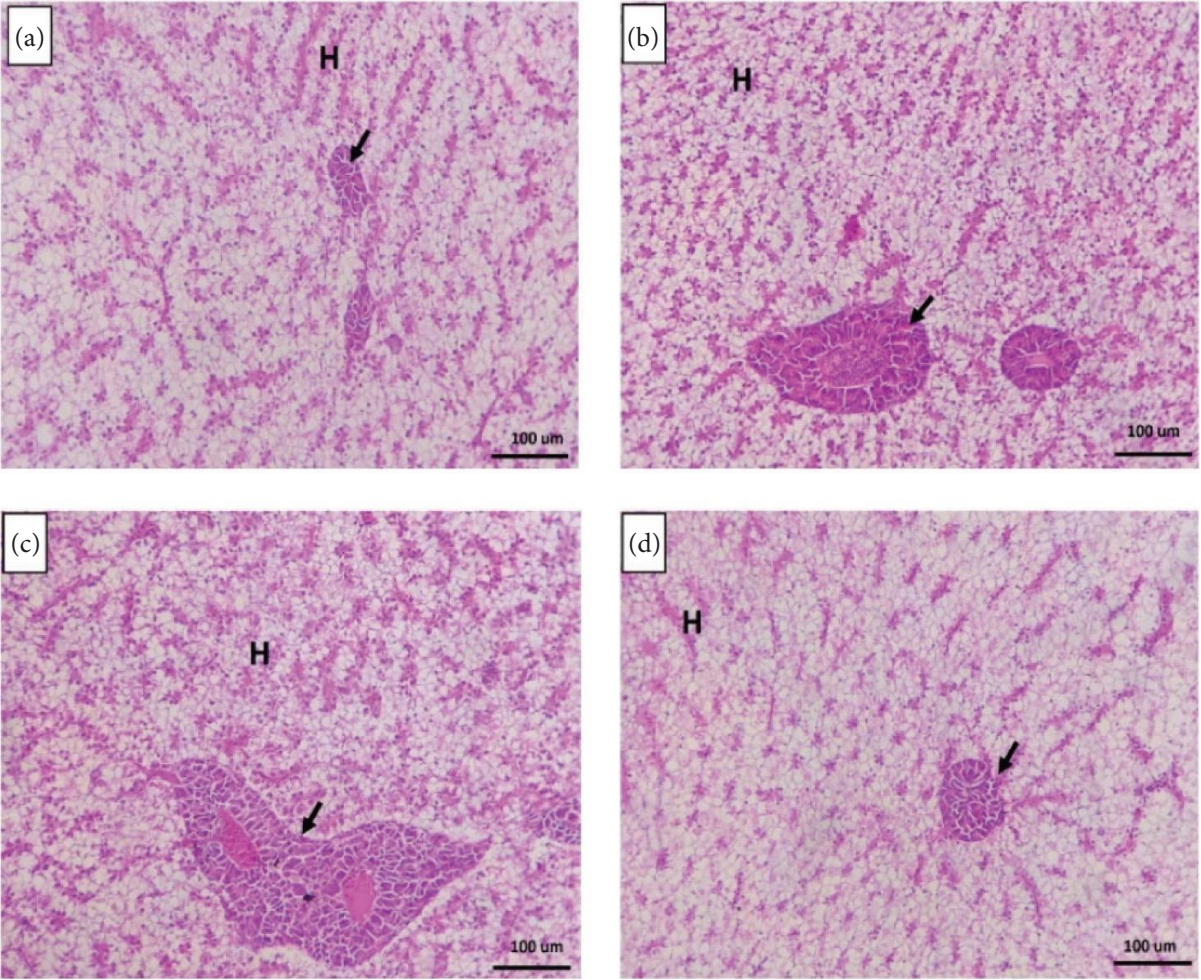
Histomicrograph showing the histological structure of the liver of Nile tilapia intestine in the fish meal (a), *T. molitor* (b), fish meal/sodium butyrate (c), and both *T. molitor*/sodium butyrate (d). The hepatic (H) and pancreatic (black arrow) structures in all groups appeared normal without deterioration or vacuolation. However, the cosupplementation with *T. molitor*/sodium butyrate stimulated an apparent glycogen deposition within the hepatocytes (d). Stain H&E. Bar 100 *µ*m.

The liver in fish-fed FM revealed normal hepatic parenchyma, intact hepatocytes, and pancreatic acinar cells (Figure 2(a)). Fish-fed *T. molitor* and SB (Figures 2(b) and 2(c)) showed similar histomorphology like FM-fed fish; however, fish fed both *T. molitor* and SB revealed improved hepatic parenchyma better than other groups. The hepatocytes showed increased glycogen deposition by forming irregular glycogen vacuoles inside their cytoplasm (Figure 2(d)).

### 3.3. Hematological Indices and Blood Biomarkers

The hematological indices of Nile tilapia-fed FM or *T. molitor* with or without SB supplementation are presented in Table 4. No marked interaction effects of protein source and dietary SB were seen on the hematological indices were detected (*P* > 0.05) except for the Hb (*P*=0.007), RBCs (*P*=0.018), and PCV (*P*=0.015) of Nile tilapia. Fish-fed FM or *T. molitor* and SB had higher Hb than those fed FM or *T. molitor* without SB. On the other hand, tilapia-fed *T. molitor* without SB had lower Hb than the other groups. The RBCs were significantly higher in tilapia-fed *T. molitor* and SB than in tilapia-fed FM with or without SB. Besides, the PCV was significantly higher in tilapia-fed FM and SB than in tilapia-fed FM without SB or *T. molitor* with or without SB.

No significant interactions were seen on the blood biomarkers (*P* > 0.05) except for the blood total protein (*P*=0.017) and globulin (*P*=0.010) (Table 5). Tilapia-fed FM or *T. molitor* with SB had higher total protein and globulin than those without dietary SB. At the same time, tilapia-fed *T. molitor* without SB had the lowest total protein and globulin levels. Only protein source was a significant factor in the ALT (*P*=0.017), creatinine (*P*=0.035), T─CHO (*P*=0.009), and TG (*P*=0.030), where these markers were higher in tilapia-fed dietary *T. molitor* than those fed FM. Dietary SB was also a significant factor in the AST level, where tilapia-fed SB had lower AST than those fed without SB (*P*=0.029).

### 3.4. Immunity and Antioxidative Responses

No marked interaction effects of protein source and dietary SB were seen on the lysozyme, CAT, and phagocytic activities, and MDA levels were detected (*P* > 0.05) in Nile tilapia (Table 6). However, dietary SB was a significant factor in the lysozyme (*P*=0.022) and phagocytic activities (*P*=0.004), where fish-fed SB had higher lysozyme and phagocytic activities than those fed without SB. Further, protein sources or dietary SB were significant factors in the CAT activity (*P*=0.030; *P*=0.004) and MDA (*P*=0.015; *P*=0.004). Fish-fed *T. molitor*, or SB had, higher CAT and lower MDA than those fed FM or without dietary SB. On the other hand, protein source and dietary SB were interactively affected by the SOD (*P*=0.033) and GPx (*P*=0.048). Fish-fed *T. molitor* and SB had higher SOD than the other groups. Besides, fish-fed FM or *T. molitor* with SB had higher GPx than the other groups. Nevertheless, tilapia-fed *T. molitor* without SB had the lowest GPx among the groups.

## 4. Discussion

Nile tilapia is a vital finfish species consumed by humanity around the globe, associated with their suitability for diverse environmental conditions and acceptability for different feeding regimes [34, 35]. Yellowworm meal has been approved as a suitable replacement for FM in aquafeed [23, 24]. In addition, sodium butyrate (SB), a functional organic acid, is involved in enhancing digestive system health and, thereby, the entire fish body's performance [52]. This study tested the addition of SB to *T. molitor*-based diets to enhance tilapias' digestion, feed utilization, and growth performance. The results showed comparable growth performance (FBW, WG, and SGR) and feed utilization (FCR and PER) in the case of tilapia-fed FM and *T. molitor* without significant differences, which agrees with Tubin et al. [36] and Anany et al. [37] who illustrate that tilapia can feed *T. molitor* up to 10% without significant difference on the growth performance and feed utilization with tilapia fed FM based diet. Interestingly, tilapia-fed *T. molitor* or FM with SB addition showed higher growth performance and improved FCR and PER than tilapia without SB addition. The lack of growth performance of tilapia-fed *T. molitor* without SB can be related to chitin, which acts as an antinutritional factor leading to low digestion capacity of fish intestines [23, 24]. The low digestibility of high inclusion levels of *T. molitor* may also result from the lack of micronutrients and the possibility of toxin 1,4-benzonchinone formation [53]. In this regard, high inclusion levels of *T. molitor* at 30%, 32.5%, 33.6%, and 30% reduced the feed utilization and growth performance of turbot (*Scophthalmus maximus*) [54], red hybrid tilapia (*Oreochromis* sp.) [55], black porgy (*Acanthopagrus schlegelii*) [56], and meager (*Argyrosomus regius*) [57], respectively. The negative impacts of *T. molitor* meal on feed digestion are closely related to the differences in fish species, feeding duration, fish sizes, and experimental conditions. For this reason, the study hypothesized that adding SB may enhance the growth performance and digestion capacity of tilapia-fed *T. molitor*, which is evident. SB is one of the salts for butyric acid formation associated with the proliferation of intestinal epithelial cells as a source of energy [33]. Further, butyric acid can eliminate the presence of harmful bacteria and activate the reproduction of beneficial bacteria in the local intestines [31]. Accordingly, fish fed with SB can show healthy intestinal features and high absorption capacity. In the same manner, Nile tilapia-fed SB revealed enhanced growth performance, as declared by Abdel-Tawwab et al. [58], Alves Jesus et al. [59], and Shalata et al. [60]. Besides, the feed utilization (FCR and PER) enhancement in tilapia-fed SB can be related to efficient digestive enzyme activity, which tightly results from SB. Although the activity of digestive enzymes was not evaluated in this study, the enhanced feed utilization is probably related to the positive role of SB in activating digestive enzymes, as stated by El-Sharkawy et al. [61] and Abdel-Tawwab et al. [58].

Fish intestines are mainly involved in the digestion and absorption of feed, which can be evaluated by detecting intestinal histological features [62]. The appearance of the intestinal villi showed a marked increase in the height and branching in the treated groups with *T. molitor* and SB along the whole length of the intestine. Similarly, Nile tilapia-fed dietary SB showed an enhanced length of intestines, intestinal muscle thickness, and villi height, as stated by Abdel-Tawwab et al. [58], Alves Jesus et al. [59], and Shalata et al. [60]. SB can support the activity of intestinal epithelial cells as a source of energy, leading to the proliferation of intestinal cells and restoration of mucosal damage [33]. The enhancement in the villi length led to increased absorption area and efficient feed utilization. Further, SB can regulate intestinal mucosal permeability and allow digested nutrients to cross into the bloodstream [31]. Thus, efficient metabolism and physiological function resulting from SB feeding may enhance growth performance and general health status [27].

Goblet cells secret mucin, which is involved in the protection of intestinal epithelium and the transport of digested nutrients through the lumen to the brush border membrane [63]. In addition, highly secreted acidic mucin is involved in the degradation of glycosidase produced by harmful bacteria [64, 65]. The results also showed an increased count for goblet cells by dietary SB when using FM or *T. molitor* as protein sources. In this regard, Dawood et al. [42] and Shalata et al. [60] reported similar results where Nile tilapia-fed dietary SB showed increased goblet cell count. However, Bai et al. [54] reported that high inclusion levels of *T. molitor* in the diets of turbot reduced the count of goblet cells. The differences in the count of goblet cells among fish species can vary due to the feeding habits, feed duration, and experimental conditions. The increased count of goblet cells in Nile tilapia-fed SB may explain the improved feed utilization in the case of using FM or *T. molitor*. These results also confirm the hypothesis of the current study that dietary SB may enhance the growth performance and well-being of Nile tilapia-fed *T. molitor* by improving intestinal digestion and, thereby, feed utilization. However, this requires further future studies to report the exact mode of action under different trial conditions.

It is well reported that the nutritional value of feeds would affect the fish's metabolic and physiological status, which can be evaluated by detecting hematological and blood biochemical indices [66]. The results showed no significant alterations of *T. molitor* on the hematological profile, while dietary SB affected the RBCs, Hb, hematocrit, and WBCs indices. Likewise, Abdel-Latif et al. [67] stated that Nile tilapia-fed SB showed elevated RBCs, Hb, hematocrit, and WBCs indices. The results confirm that dietary *T. molitor* is safe for tilapia, and dietary SB is needed to enhance the metabolic and physiological function of Nile tilapia. The results also showed elevated blood protein, globulin, and albumin in Nile tilapia-fed SB, either with or without *T. molitor*. The results are in line with Abdel-Latif et al. [67] and Dawood et al. [42], who reported enhanced blood proteins in the Nile tilapia-fed dietary SB. Increased blood protein, WBCs, RBCs, Hb, and hematocrit in Nile tilapia by dietary SB are tightly correlated with the improvements in the feed metabolism and availability of nutrients for physiological and immunological responses. Further, the absence of hepatic and kidney failure function, as shown by ALT, AST, urea, and creatinine levels, confirms the nutritional balance and safe use of mealworms and SB in tilapia feeds. This enhancement in the health status may explain the improved growth performance of Nile tilapia-fed *T. molitor* and SB.

In addition to the hematological and biochemical blood indices, antioxidative status and immune response were evaluated in Nile tilapia-fed *T. molitor* and SB. The results indicated elevated SOD and GPx in Nile tilapia by dietary *T. molitor* and SB. Nonetheless, no marked effects on the MDA levels indicate the absence of oxidative stress resulting from mealworms and SB feeding. Similarly, Anany et al. [37], Sánchez-Muros et al. [38], and Zhang et al. [19] indicated that dietary *T. molitor* activated the antioxidative status by increasing the SOD and GPx in Nile tilapia. Further, Monier et al. [68] and Dawood et al. [42] indicated enhanced SOD and GPx in the Nile tilapia-fed dietary SB. Concisely, the study showed enhanced lysozyme and phagocytic activities in Nile tilapia by dietary SB, while *T. molitor* did not affect the immunity of fish. Similarly, Abdel-Latif et al. and El-Sharkawy et al. [61] reported enhanced lysozyme and phagocytic activities in Nile tilapia by dietary SB. The enhancement of immunity and antioxidative status of Nile tilapia-fed *T. molitor* and SB suggest this feeding strategy to improve the capacity to resist biotic and abiotic stressors that may occur during the farming season. In addition, mealworms are rich in chitin and *β*-carotene contents, which is well known for antioxidative and immunostimulant roles since chitin can scavenge free radicals involved in oxidative stress [69, 70]. Further, chitin has specific pattern-recognition receptors that stimulate the immune system [71]. Therefore, the potential roles of chitin as antioxidative and immunostimulant agents have been well investigated in aquatic animals [72, 73].

Lately, in accordance with the outputs of the current study, Anany et al. [37] reported that dietary *S. cerevisiae* could enhance the zootechnical digestion capacity and health status of Nile tilapia-fed dietary *T. molitor*. Our team planned those two studies as a trial for finding sustainable solutions for alternative protein ingredients for Nile tilapia aquaculture without affecting production and well-being. Further, to overcome the undesirable effects of *T. molitor* on Nile tilapia's digestibility and health through functional additives such as probiotics and organic acids. Still, more effort is required to investigate sustainable approaches and low-cost, high-quality ingredients for continuous Nile tilapia production.

## 5. Conclusion

In conclusion, replacing FM with *T. molitor* did not compromise the growth performance, feed utilization, intestinal health, blood markers, and immunity of Nile tilapia. Further, the addition of sodium butyrate can markedly enhance the growth performance, digestion capacity, intestinal histological features, and antioxidative and immune responses of Nile tilapia. Hence, adding sodium butyrate when using mealworms as a replacer for FM in tilapia feeding is highly recommended. Further future efforts are needed to test different nontraditional ingredients for tilapia and deep application for functional additives to sustain tilapia productivity globally.

## Figures and Tables

**Table 1:** Formulation and composition of the basal diet.

Ingredients (%)	Fish meal		*T. molitor*	
	─SB	+SB	─SB	+SB
Fish meal (65% CP)	10	10	0	0
*T. molitor* meal (60% CP)	0	0	10	10
Soybean meal (44% CP)	32	32	33	33
Sodium butyrate (SB)	0	0.1	0	0.1
Yellow corn meal	18	18	18	18
Corn gluten	6	6	6	6
Wheat bran	18	18	18	18
Rice bran	7	7	7	7
Wheat flour	5.25	5.15	5.25	5.15
Fish oil	2	2	1	1
Corn oil	1	1	1	1
Vitamin and mineral mix^1^	0.5	0.5	0.5	0.5
Dicalcium phosphate	0.2	0.2	0.2	0.2
Vitamin C	0.05	0.05	0.05	0.05
Methionine	0	0	0.002	0.002
Lysine	0	0	0.005	0.005
Total	100	100	100	100
Chemical composition				
Crude protein (%)	30.60	30.59	30.12	30.11
Crude lipids (%)	5.60	5.60	6.54	6.54
Ash (%)	5.11	5.09	5.21	5.14
Fibers (%)	4.62	4.52	4.62	4.36
Gross energy (MJ/kg)^2^	18.73	18.75	18.90	18.95
P/E ratio^3^	16.33	16.31	15.94	15.89
Essential amino acid (g/kg)				
Arginine	1.81	1.99	1.68	1.69
Histidine	1.09	1.09	0.93	0.93
Isoleucine	1.60	1.60	1.32	1.33
Leucine	3.76	3.76	3.28	3.28
Lysine	1.74	1.74	1.23	1.24
Methionine	0.76	0.76	0.58	0.58
Phenylalanine	1.89	1.89	1.64	1.64
Threonine	1.39	1.39	1.11	1.12
Tryptophan	0.36	0.36	0.30	0.30
Valine	1.78	1.78	1.45	1.45

^1^The mixture of vitamins and minerals was added, according to Anany et al. [37]. ^2^Gross energy (GE) was calculated based on protein, lipid, and carbohydrate values as 23.6, 39.5, and 17.2 kJ/g, respectively, NRC [40]. ^3^Protein to energy ratio (P/E) ratio (mg CP/kJ GE) = CP/GE × 1,000 (kJ/100 g diet).

**Table 2:** Growth performance of Nile tilapia fed test diets for 60 days.

	IW (g)	FW (g)	WG (%)	SGR	FI	FCR	PER	Survival (%)
Fish meal	6.03	21.95^b^	263.79^b^	2.15^b^	22.47	1.41^b^	2.33^a^	98.33
Fish meal + SB	6.07	23.07^a^	280.44^a^	2.23^a^	23.45	1.39^b^	2.38^a^	98.33
*T. molitor*	6.02	20.63^b^	242.79^b^	2.05^b^	22.53	1.54^a^	2.12^b^	96.67
*T. molitor* + SB	6.05	22.17^ab^	266.58^ab^	2.16^b^	21.57	1.34^b^	2.44^a^	96.67
Pooled SE	0.02	0.30	5.21	0.04	0.28	0.03	0.05	0.97
Main effect								
Protein source								
Fish meal	6.05	22.51	272.12	2.19	22.96	1.40	2.35	98.33
* T. molitor*	6.03	21.40	254.68	2.11	22.05	1.44	2.28	96.67
Sodium butyrate (SB)								
─SB	6.03	21.29	253.29	2.10	22.50	1.48	2.23	97.50
+SB	6.06	22.62	273.51	2.20	22.51	1.36	2.41	97.50
Two-way ANOVA (*P*-value)								
Protein source	0.799	0.013	0.052	0.042	0.078	0.328	0.31	0.471
SB	0.554	0.005	0.029	0.027	0.986	0.018	0.022	1
Protein source × SB	1	0.013	0.012	0.003	0.062	0.044	0.017	1

Values are an average of three replicates. Different letters indicate significant differences (*P* < 0.05) between dietary protein sources (fish meal or *T. molitor* meal) and sodium butyrate by Tukey's test when significant interactions are seen at (*P* < 0.05). However, in case of nonsignificant interactions at (*P* > 0.05) between the main effects between dietary protein sources (fish meal or *T. molitor* meal) or sodium butyrate, respectively by *t*-test at (*P* < 0.05). IW: initial weight, FW: final weight, WG: weight gain, SGR: specific growth rate, FCR: feed conversion ratio, PER: protein efficiency ratio.

**Table 3:** Intestinal morphometry of Nile tilapia fed test diets for 60 days.

	Villus height (*μ*m)	Villus width (*μ*m)	Crypt depth (*μ*m)	Muscularis thickness (*μ*m)	Goblet cell count
Fish meal	122.21^d^	85.91^b^	42.31^c^	3.33^d^	22.47^b^
Fish meal + SB	504.73^a^	103.6^a^	71.41^a^	13.05^b^	23.45^a^
*T. molitor*	216.47^c^	84.18^b^	57.87^b^	7.33^c^	22.53^b^
*T. molitor* + SB	442.33^b^	108.58^a^	75.67^a^	28.67^a^	23.57^a^
Pooled SE	12.36	4.59	3.11	0.67	1.32
Main effect					
Protein source					
Fish meal	313.47	94.76	56.86	8.17	22.96
* T. molitor*	329.40^*∗*^	96.38	66.77^*∗*^	18.00^*∗*^	23.05
Sodium butyrate (SB)					
─SB	169.34	85.05	50.09	5.33	22.50
+SB	473.53^#^	106.09^#^	73.54^#^	20.84^#^	23.51^#^
Two-way ANOVA (*P*-value)					
Protein source	0.003	0.211	0.005	0.024	0.032
SB	0.001	0.002	0.031	0.035	0.011
Protein source × SB	0.021	0.043	0.003	0.002	0.003

Values are an average of three replicates. Different letters indicate significant differences (*P* < 0.05) between dietary protein sources (fish meal or *T*. *molitor* meal) and sodium butyrate by Tukey's test when significant interactions are seen at (*P* < 0.05). However, in case of nonsignificant interactions at (*P* > 0.05) between the main effects, the symbols of ( ^*∗*^) and (^#^) are used to refer to the significances between dietary protein sources (fish meal or *T. molitor* meal) or sodium butyrate, respectively, by *t*-test at (*P* < 0.05).

**Table 4:** Hematological indices of Nile tilapia fed test diets for 60 days.

	Hb (g/100 mL)	RBCs (×10^6^/mm^3^)	PCV (%)	MCV (*µ*m^3^/cell)	MCH (pg/cell)	MCHC (%)	WBCs (×10^3^/mm^3^)	Heterophils (%)	Lym (%)	Mon (%)	Eosinophils (%)	Basophil (%)
Fish meal	11.33^b^	3.56^b^	37.33^b^	106.69	32.18	30.34	9.96	16.33	73.00	9.00	0.67	1.00
Fish meal + SB	12.40^a^	3.93^b^	42.00^a^	108.51	31.90	29.52	10.63	13.33	76.67	8.00	1.00	1.00
*T. molitor*	10.73^c^	4.07^ab^	39.00^b^	95.86	26.41	27.54	9.78	14.33	76.33	7.67	1.00	0.67
*T. molitor* + SB	12.60^a^	4.13^a^	40.67^b^	98.40	30.52	31.01	10.77	11.00	79.67	8.33	0.67	0.33
Pooled SE	0.28	0.13	0.66	3.40	0.97	0.51	0.17	1.36	1.34	0.35	0.17	0.13
Main effect												
Protein source												
Fish meal	11.87	3.75	39.67	107.60	32.04	29.93	10.30	14.83	74.83	8.50	0.83	1.00
* T. molitor*	11.67	4.10	39.83	97.13	28.46	29.28	10.28	12.67	78.00	8.00	0.83	0.50
Sodium butyrate (SB)												
─SB	11.03	3.82	38.17	101.27	29.30	28.94	9.87	15.33	74.67	8.33	0.83	0.83
+SB	12.50	4.03	41.33	103.45	31.21	30.27	10.70	12.17	78.17	8.17	0.83	0.67
Two-way ANOVA (*P*-value)												
Protein source	0.607	0.195	0.864	0.175	0.053	0.422	0.940	0.474	0.268	0.521	1.000	0.067
SB	0.004	0.422	0.010	0.764	0.259	0.124	0.018	0.305	0.225	0.829	1.000	0.500
Protein source × SB	0.007	0.018	0.015	0.960	0.202	0.124	0.584	0.955	0.952	0.296	0.397	0.500

Values are an average of three replicates. Different letters indicate significant differences (*P* < 0.05) between dietary protein sources (fish meal or *T*. *molitor* meal) and sodium butyrate by Tukey's test when significant interactions are seen at (*P* < 0.05). However, in case of nonsignificant interactions at (*P* > 0.05) between the main effects between dietary protein sources (fish meal or *T*. *molitor* meal) or sodium butyrate, respectively by *t*-test at (*P* < 0.05). Hb: hemoglobin concentration, RBC: red blood cell, PCV: packed cell volume, MCV: mean corpuscular volume, MCH: mean corpuscular hemoglobin, MCHC: mean corpuscular hemoglobin concentration, WBC: white blood cell, Lym: lymphocytes, Mon: monocytes.

**Table 5:** Blood biochemical indices of Nile tilapia fed test diets for 60 days.

	ALT (U/L)	AST (U/L)	Total protein (g/dL)	Albumin (g/dL)	Globulin (g/dL)	Creatinine (mg/dL)	T-CHO (mg/dL)	TG (mg/dL)
Fish meal	18.39	22.13	4.51^b^	1.50	3.01^b^	0.26	69.04	92.70
Fish meal + SB	15.16	19.91	4.89^a^	1.60	3.29^a^	0.26	89.68	101.34
*Tenebrio molitor*	21.85	24.53	4.22^c^	1.58	2.64^c^	0.31	90.71	105.71
*Tenebrio molitor* + SB	20.36	17.42	4.81^a^	1.58	3.22^a^	0.28	76.91	99.76
Pooled SE	0.97	1.09	0.11	0.03	0.09	0.01	3.70	1.86
Main effect								
Protein source								
Fish meal	16.78	21.02	4.70	1.55	3.15	0.26	79.36	97.02
* Tenebrio molitor*	21.10^*∗*^	20.98	4.51	1.58	2.93	0.30^*∗*^	83.81^*∗*^	102.74^*∗*^
Sodium butyrate (SB)								
─SB	20.12	23.33^#^	4.55	1.59	2.96	0.29	79.88	99.21
+SB	17.76	18.67	4.66	1.54	3.12	0.27	83.29	100.55
Two-way ANOVA (*P*-value)								
Protein source	0.017	0.982	0.279	0.632	0.129	0.035	0.009	0.030
SB	0.142	0.029	0.535	0.469	0.272	0.428	0.576	0.650
Protein source × SB	0.565	0.201	0.017	0.440	0.010	0.428	0.119	0.134

Values are an average of three replicates. Different letters indicate significant differences (*P* < 0.05) between dietary protein sources (fish meal or *T. molitor* meal) and sodium butyrate by Tukey's test when significant interactions are seen at (*P* < 0.05). However, in case of nonsignificant interactions at (*P* > 0.05) between the main effects, the symbols of ( ^*∗*^) and (^#^) are used to refer to the significances between dietary protein sources (fish meal or *T. molitor* meal) or sodium butyrate, respectively by *t*-test at (*P* < 0.05). ALT: alanine aminotransferase, AST: aspartate aminotransferase, T-CHO: total cholesterol, TG: triglycerides.

**Table 6:** Blood immunity and antioxidative responses of Nile tilapia fed test diets for 60 days.

	Lysozyme activity (unit/mL)	Phagocytic activity (%)	Phagocytic index	Superoxide dismutase (IU/L)	Catalase (IU/L)	Glutathione peroxidase (IU/L)	Malondialdehyde (nmol/mL)
Fish meal	3.45	8.79	1.14	9.19^b^	6.04	9.78^b^	21.04
Fish meal + SB	4.13	9.88	1.09	9.84^ab^	6.28	10.76^a^	18.49
*T. molitor*	3.38	7.08	0.95	9.51^b^	7.59	8.25^c^	17.30
*T. molitor* + SB	4.49	7.42	1.15	10.42^a^	8.36	10.44^a^	16.31
Pooled SE	0.20	0.35	0.04	0.19	0.41	0.45	0.84
Main effect							
Protein source							
Fish meal	3.79	9.33^*∗*^	1.12	9.52	6.16	10.27	19.77^*∗*^
* T. molitor*	3.94	7.25	1.05	9.96	7.98^*∗*^	9.35	16.80
Sodium butyrate (SB)							
─SB	3.41	7.93	1.05	9.81	6.82	9.02	19.17^#^
+SB	4.31^#^	8.65^#^	1.12	9.67	7.32^#^	10.60	17.40
Two-way ANOVA (*P*-value)							
Protein source	0.659	0.001	0.355	0.180	0.030	0.289	0.015
SB	0.022	0.004	0.312	0.675	0.004	0.089	0.004
Protein source × SB	0.523	0.067	0.110	0.033	0.710	0.048	0.618

Values are an average of three replicates. Different letters indicate significant differences (*P* < 0.05) between dietary protein sources (fish meal or *T. molitor* meal) and sodium butyrate by Tukey's test when significant interactions are seen at (*P* < 0.05). However, in case of nonsignificant interactions at (*P* > 0.05) between the main effects, the symbols of ( ^*∗*^) and (^#^) are used to refer to the significances between dietary protein sources (fish meal or *T. molitor* meal) or sodium butyrate, respectively, by *t*-test at (*P* < 0.05).

## Data Availability

All other relevant data are available from the corresponding authors upon reasonable request.

## References

[1] Bartley D. M., Beveridge M. C. M., Phillips M. J., Tacon A. G. J., Verdegem M. (2023). Enhancing the nutritional values of farmed fish production systems. *Reviews in Aquaculture*.

[2] FAO (2022). *The State of World Fisheries and Aquaculture 2022*.

[3] Colombo S. M., Roy K., Mraz J. (2022). Towards achieving circularity and sustainability in feeds for farmed blue foods. *Reviews in Aquaculture*.

[4] Mugwanya M., Dawood M. A. O., Kimera F., Sewilam H. (2023). Replacement of fish meal with fermented plant proteins in the aquafeed industry: a systematic review and meta-analysis. *Reviews in Aquaculture*.

[5] Somdare P. O., Hamid N. K. A., Kari Z. A. (2023). Effect of papaya leaf extract inclusion on growth performance and haematological parameters of red hybrid tilapia, *Oreochromis mossambicus* × *Oreochromis niloticus* fed diets formulated with Hermetia meal and Azolla. *Agriculture Reports*.

[6] Midhun S. J., Arun D., Mathew J., Jose M. S., Kumar R. E. K. A. (2023). Chapter 12—alternative feed technology in aquaculture. *Recent Advances in Aquaculture Microbial Technology*.

[7] Dawood M. A. O., Habotta O. A. E., Elsabagh M. (2022). Fruit processing by-products in the aquafeed industry: a feasible strategy for aquaculture sustainability. *Reviews in Aquaculture*.

[8] Gasco L., Biasato I., Enes P., Gai F., Morales-Ramos J. A., Rojas M. G., Shapiro-Ilan D. I. (2023). Chapter 16—Potential and challenges for the use of insects as feed for aquaculture. *Mass Production of Beneficial Organisms*.

[9] Tippayadara N., Dawood M. A. O., Krutmuang P., Hoseinifar S. H., Doan H. V., Paolucci M. (2021). Replacement of fish meal by black soldier fly (*Hermetia illucens*) larvae meal: effects on growth, haematology, and skin mucus immunity of Nile tilapia, *Oreochromis niloticus*. *Animals*.

[10] Kok B., Malcorps W., Tlusty M. F. (2020). Fish as feed: using economic allocation to quantify the fish in : fish out ratio of major fed aquaculture species. *Aquaculture*.

[11] Gasco L., Acuti G., Bani P. (2020). Insect and fish by-products as sustainable alternatives to conventional animal proteins in animal nutrition. *Italian Journal of Animal Science*.

[12] Iliya I., Obaroh I. O., Ukatu V. E., Bshar U. D. (2023). Evaluation of some agricultural bye-product as floaters in fish feed formulation. *Journal of Advanced Education and Sciences*.

[13] Gasco L., Gai F., Maricchiolo G., Gasco L., Gai F., Maricchiolo G. (2018). Fishmeal alternative protein sources for aquaculture feeds. *Feeds for the Aquaculture Sector: Current Situation and Alternative Sources*.

[14] Maulu S., Langi S., Hasimuna O. J. (2022). Recent advances in the utilization of insects as an ingredient in aquafeeds: a review. *Animal Nutrition*.

[15] Alfiko Y., Xie D., Astuti R. T., Wong J., Wang L. (2022). Insects as a feed ingredient for fish culture: status and trends. *Aquaculture and Fisheries*.

[16] Shafique L., Abdel-Latif H. M. R., Hassan F.-u. (2021). The feasibility of using yellow mealworms (*Tenebrio molitor*): towards a sustainable aquafeed industry. *Animals*.

[17] Hasan I., Gai F., Cirrincione S., Rimoldi S., Saroglia G., Terova G. (2023). Chitinase and insect meal in aquaculture nutrition: a comprehensive overview of the latest achievements. *Fishes*.

[18] Khanal P., Pandey D., Næss G. (2023). Yellow mealworms (*Tenebrio molitor*) as an alternative animal feed source: a comprehensive characterization of nutritional values and the larval gut microbiome. *Journal of Cleaner Production*.

[19] Zhang L., Wu H.-X., Li W.-J. (2023). Partial replacement of soybean meal by yellow mealworm (*Tenebrio molitor*) meal influences the flesh quality of Nile tilapia (*Oreochromis niloticus*). *Animal Nutrition*.

[20] Li H., Xue R., Sun J., Ji H. (2023). Improving flesh quality of grass carp (*Ctenopharyngodon idellus*) by completely replacing dietary soybean meal with yellow mealworm (*Tenebrio molitor*). *Animal Nutrition*.

[21] Tran H. Q., Prokešová M., Zare M. (2022). Production performance, nutrient digestibility, serum biochemistry, fillet composition, intestinal microbiota and environmental impacts of European perch (*Perca fluviatilis*) fed defatted mealworm (*Tenebrio molitor*). *Aquaculture*.

[22] Tran H. Q., Nguyen T. T., Prokešová M. D. (2023). Insight into bioavailability of various insect meals for European perch (*Perca fluviatilis*): a nutritional and stable isotopic evaluation. *Aquaculture*.

[23] Zhang J., Dong Y., Song K. (2022). Effects of the replacement of dietary fish meal with defatted yellow mealworm (*Tenebrio molitor*) on juvenile large yellow croakers (*Larimichthys crocea*) growth and gut health. *Animals*.

[24] Henry M. A., Gai F., Enes P., Peréz-Jiménez A., Gasco L. (2018). Effect of partial dietary replacement of fishmeal by yellow mealworm (*Tenebrio molitor*) larvae meal on the innate immune response and intestinal antioxidant enzymes of rainbow trout (*Oncorhynchus mykiss*). *Fish & Shellfish Immunology*.

[25] Hasan I., Rimoldi S., Saroglia G., Terova G. (2023). Sustainable fish feeds with insects and probiotics positively affect freshwater and marine fish gut microbiota. *Animals*.

[26] Wang B., Thompson K. D., Wangkahart E. (2022). Strategies to enhance tilapia immunity to improve their health in aquaculture. *Reviews in Aquaculture*.

[27] Dawood M. A. O. (2021). Nutritional immunity of fish intestines: important insights for sustainable aquaculture. *Reviews in Aquaculture*.

[28] Xun P., Zhou C., Huang X. (2023). Effects of dietary sodium acetate on intestinal health of juvenile *Trachinotus ovatus* based on multi-omics approach. *Aquaculture*.

[29] Abdel-Latif H. M. R., Abdel-Tawwab M., Dawood M. A. O., Menanteau-Ledouble S., El-Matbouli M. (2020). Benefits of dietary butyric acid, sodium butyrate, and their protected forms in aquafeeds: a review. *Reviews in Fisheries Science & Aquaculture*.

[30] Mehrgan M. S., Shekarabi S. P. H., Azari A., Yilmaz S., Lückstädt C., Islami H. R. (2022). Synergistic effects of sodium butyrate and sodium propionate on the growth performance, blood biochemistry, immunity, and immune-related gene expression of goldfish (*Carassius auratus*). *Aquaculture International*.

[31] Fabay R. V., Serrano A. E., Alejos M. S., Fabay J. V. (2022). Effects of dietary acidification and acid source on fish growth and feed efficiency (review). *World Academy of Sciences Journal*.

[32] Ahsan U., Cengiz Ö., Raza I. (2016). Sodium butyrate in chicken nutrition: the dynamics of performance, gut microbiota, gut morphology, and immunity. *World’s Poultry Science Journal*.

[33] Oyabambi A. O., Olaniyi K. S. (2023). Sodium butyrate aggravates glucose dysregulation and dyslipidemia in high fat-fed Wistar rats. *Metabolism Open*.

[34] Adeshina I., Akpoilih B. U., Udom B. F., Adeniyi O. V., Abdel-Tawwab M. (2023). Interactive effects of dietary phosphorus and microbial phytase on growth performance, intestinal morphometry, and welfare of Nile tilapia (*Oreochromis niloticus*) fed on low-fishmeal diets. *Aquaculture*.

[35] Xiong W., Guo C., Gozlan R. E., Liu J. (2023). Tilapia introduction in China: economic boom in aquaculture versus ecological threats to ecosystems. *Reviews in Aquaculture*.

[36] Tubin J. S. B., Paiano D., Hashimoto G. S. (2020). *Tenebrio molitor* meal in diets for Nile tilapia juveniles reared in biofloc system. *Aquaculture*.

[37] Anany E. M., Ibrahim M. A., El-Razek I. M. A. (2023). Combined effects of yellow mealworm (*Tenebrio molitor*) and *Saccharomyces cerevisiae* on the growth performance, feed utilization intestinal health, and blood biomarkers of Nile tilapia (*Oreochromis niloticus*) fed fish meal-free diets. *Probiotics and Antimicrobial Proteins*.

[38] Sánchez-Muros M. J., de Haro C., Sanz A., Trenzado C. E., Villareces S., Barroso F. G. (2016). Nutritional evaluation of *Tenebrio molitor* meal as fishmeal substitute for tilapia (*Oreochromis niloticus*) diet. *Aquaculture Nutrition*.

[39] NRC (2011). *Nutrient Requirements of Fish and Shrimp*.

[40] Bancroft J., Stevens A., Turner D. (1996). *Theory and Practice of Histological Techniques*.

[41] AOAC (2012). *Official Methods of Analysis of AOAC International*.

[42] Dawood M. A. O., Eweedah N. M., Elbialy Z. I., Abdelhamid A. I. (2020). Dietary sodium butyrate ameliorated the blood stress biomarkers, heat shock proteins, and immune response of Nile tilapia (*Oreochromis niloticus*) exposed to heat stress. *Journal of Thermal Biology*.

[43] Gewaily M. S., Abumandour M. M. A. (2021). Gross morphological, histological and scanning electron specifications of the oropharyngeal cavity of the hooded crow (*Corvus cornix pallescens*). *Anatomia, Histologia, Embryologia*.

[44] Houston A. (1990). *Blood and Circulation/Methods for Fish Biology*.

[45] Blaxhall P. C., Daisley K. W. (1973). Routine haematological methods for use with fish blood. *Journal of Fish Biology*.

[46] Doumas B. T., Bayse D. D., Carter R. J., Peters T., Schaffer R. (1981). A candidate reference method for determination of total protein in serum. I. Development and validation. *Clinical Chemistry*.

[47] Dumas B. T., Biggs H. G. (1972). *Standard Methods of Clinical Chemistry*.

[48] Ellis A., Stolen J., Fletcher T., Anderson D., Robertson B., Van Muiswinkel W. (1990). Lysozyme assay in techniques in fish immunology. *Technique in Fish Immunology*.

[49] Cai W.-Q., Li S.-F., Ma J.-Y. (2004). Diseases resistance of Nile tilapia (*Oreochromis niloticus*), blue tilapia (*Oreochromis aureus*) and their hybrid (female Nile tilapia × male blue tilapia) to *Aeromonas sobria*. *Aquaculture*.

[50] Kawahara E., Ueda T., Nomura S. (1991). In vitro phagocytic activity of white-spotted char blood cells after injection with *Aeromonas salmonicida* extracellular products. *Fish Pathology*.

[51] Mihara M., Uchiyama M. (1978). Determination of malonaldehyde precursor in tissues by thiobarbituric acid test. *Analytical Biochemistry*.

[52] Zhang J., Zhong L., Chi S., Chu W., Liu Y., Hu Y. (2020). Sodium butyrate supplementation in high-soybean meal diets for juvenile rice field eel (*Monopterus albus*): effects on growth, immune response and intestinal health. *Aquaculture*.

[53] Vargas-Abúndez A. J., Randazzo B., Foddai M. (2019). Insect meal based diets for clownfish: biometric, histological, spectroscopic, biochemical and molecular implications. *Aquaculture*.

[54] Bai N., Li Q., Pan S., Qi Z., Deng W., Gu M. (2023). Effects of defatted yellow mealworm (*Tenebrio molitor*) on growth performance, intestine, and liver health of turbot (*Scophthalmus maximus*). *Animal Feed Science and Technology*.

[55] Zainab-L I., Ng W. K., Sudesh K. (2022). Potential of mealworms used in polyhydroxyalkanoate/bioplastic recovery as red hybrid tilapia (*Oreochromis* sp.) feed ingredient. *Scientific Reports*.

[56] Jeong S.-M., Khosravi S., Kim K.-W. (2022). Potential of mealworm, *Tenebrio molitor*, meal as a sustainable dietary protein source for juvenile black porgy, *Acanthopagrus schlegelii*. *Aquaculture Reports*.

[57] Coutinho F., Castro C., Guerreiro I. (2021). Mealworm larvae meal in diets for meagre juveniles: growth, nutrient digestibility and digestive enzymes activity. *Aquaculture*.

[58] Abdel-Tawwab M., Shukry M., Farrag F. A., El-Shafai N. M., Dawood M. A. O., Abdel-Latif H. M. R. (2021). Dietary sodium butyrate nanoparticles enhanced growth, digestive enzyme activities, intestinal histomorphometry, and transcription of growth-related genes in Nile tilapia juveniles. *Aquaculture*.

[59] Jesus G. F. Alves, Owatari M. S., Pereira S. A. (2021). Effects of sodium butyrate and Lippia origanoides essential oil blend on growth, intestinal microbiota, histology, and haemato-immunological response of Nile tilapia. *Fish & Shellfish Immunology*.

[60] Shalata H. A., Bahattab O., Zayed M. M. (2021). Synergistic effects of dietary sodium butyrate and *Spirulina platensis* on growth performance, carcass composition, blood health, and intestinal histomorphology of Nile tilapia (*Oreochromis niloticus*). *Aquaculture Reports*.

[61] El-Sharkawy E. A., El-Razek I. M. A., Amer A. A. (2023). Effects of sodium butyrate on the growth performance, digestive enzyme activity, intestinal health, and immune responses of Thinlip Grey Mullet (*Liza ramada*) juveniles. *Aquaculture Reports*.

[62] Fang L., Wang Q., Guo X., Pan X., Li X. (2021). Effects of dietary sodium butyrate on growth performance, antioxidant capacity, intestinal histomorphology and immune response in juvenile Pengze crucian carp (*Carassius auratus* Pengze). *Aquaculture Reports*.

[63] Faure M., Mettraux C., Moennoz D. (2006). Specific amino acids increase mucin synthesis and microbiota in dextran sulfate sodium-treated rats. *The Journal of Nutrition*.

[64] Barca A., Abramo F., Nazerian S. (2023). Hermetia illucens for replacing fishmeal in aquafeeds: effects on fish growth performance, intestinal morphology, and gene expression in the zebrafish (*Danio rerio*) model. *Fishes*.

[65] Johansson M. E. V., Sjövall H., Hansson G. C. (2013). The gastrointestinal mucus system in health and disease. *Nature Reviews Gastroenterology & Hepatology*.

[66] López L. M., Flores-Ibarra M., Bañuelos-Vargas I., Galaviz M. A., True C. D. (2015). Effect of fishmeal replacement by soy protein concentrate with taurine supplementation on growth performance, hematological and biochemical status, and liver histology of totoaba juveniles (*Totoaba macdonaldi*). *Fish Physiology and Biochemistry*.

[67] Abdel-Latif H. M. R., Hendam B. M., Shukry M. (2021). Effects of sodium butyrate nanoparticles on the hemato-immunological indices, hepatic antioxidant capacity, and gene expression responses in *Oreochromis niloticus*. *Fish & Shellfish Immunology*.

[68] Monier M. N., El-Naby A. S. Abd, Samir F., Abdel-Tawwab M. (2022). Positive effects of dietary nanosized sodium butyrate on growth performance, immune, antioxidant indices, and resistance of Nile tilapia to waterborne copper toxicity. *Aquaculture Reports*.

[69] Hamed I., Özogul F., Regenstein J. M. (2016). Industrial applications of crustacean by-products (chitin, chitosan, and chitooligosaccharides): a review. *Trends in Food Science & Technology*.

[70] Park B. K., Kim M.-M. (2010). Applications of chitin and its derivatives in biological medicine. *International Journal of Molecular Sciences*.

[71] Rajoka M. S. R., Mehwish H. M., Wu Y. (2020). Chitin/chitosan derivatives and their interactions with microorganisms: a comprehensive review and future perspectives. *Critical Reviews in Biotechnology*.

[72] Mohan K., Rajan D. K., Ganesan A. R., Divya D., Johansen J., Zhang S. (2023). Chitin, chitosan and chitooligosaccharides as potential growth promoters and immunostimulants in aquaculture: a comprehensive review. *International Journal of Biological Macromolecules*.

[73] Kamilya D., Khan M. I. R., Gopi S., Thomas S., Pius A. (2020). Chapter 24—chitin and chitosan as promising immunostimulant for aquaculture. *Handbook of Chitin and Chitosan*.

